# Borderline Personality Disorder and Autism Spectrum Disorder: A Case Report

**DOI:** 10.1192/j.eurpsy.2025.1916

**Published:** 2025-08-26

**Authors:** R. F. Dionísio, C. Portela, D. Areias, S. M. Sousa, M. F. Santos, C. Fonseca

**Affiliations:** 1 Hospital de Magalhães Lemos, Unidade Local de Saúde de Santo António, Porto, Portugal

## Abstract

**Introduction:**

Autism spectrum disorders (ASD) are underdiagnosed in women due both to diagnostic bias, and the quieter, less visible signs and symptoms of female autism. Distress generally only becomes manifest during mid childhood and adolescence, when mental illness gets misidentified as the primary cause. The symptomatic overlap of ASD and Borderline Personality Disorder (BPD) has been noted and at the cognitive level, ASD includes difficulties in reading others emotions and core cognitive features of BPD also include altered social cognition.

**Objectives:**

The aims of this paper are to present a case report of a patient diagnosed with BPD and ASD and to provide a summary of the current literature concerning this double diagnosis.

**Methods:**

A description of the case is made and relevant articles were identified by searching the following terms: “borderline personality disorder” and “autism/autism spectrum disorders”. Research was restricted to articles concerning humans and published between 2014 and 2024 in English.

**Results:**

The case report describes a young female of 19 years old that was evaluated in an emergency context due to suicidal ideation and was then referred to both psychiatry and psychology. She was diagnosed with BPD and ASD in adolescence while she was seeing a child psychiatrist. In the consultations she reports marked emotional dysregulation, irritability, difficulties in tolerating some textures and increased sensitivity to noise and light, as well as frequent worries about peer relationships and self-image with a need to do things differently to fit in.

Despite some controversial results and lack of homogeneity in the methods used for the diagnostic assessment, subjects with BPD reported higher scores on tests evaluating the presence of ASD compared to a non-clinical population and hypothesized the presence of unrecognized ASD in some BPD patients or vice versa, while also describing a shared vulnerability towards traumatic events.

**Conclusions:**

This case illustrates a common and complex clinical challenge, where the double doses of emotional dysregulation and disrupted social interchange overwhelm either ASD or BPD approaches considered separately. Diagnostic differentiation is crucial toward targeted therapeutic interventions (psychopharmacological and psychosocial). It is still difficult to draw accurate conclusions based on the recent literature. Research in sex/gender difference is still limited, with heterogeneous results, and further studies are needed to enlighten the clinical similarities and diagnostic overlap between ASD females and BPD.

**Disclosure of Interest:**

None Declared

